# Association between Sedentary Behaviour and Physical, Cognitive, and Psychosocial Status among Older Adults in Assisted Living

**DOI:** 10.1155/2017/9160504

**Published:** 2017-08-24

**Authors:** Pet-Ming Leung, Andreas Ejupi, Kimberley S. van Schooten, Omar Aziz, Fabio Feldman, Dawn C. Mackey, Maureen C. Ashe, Stephen N. Robinovitch

**Affiliations:** ^1^Department of Biomedical Physiology and Kinesiology, Simon Fraser University, Burnaby, BC, Canada V5A 1S6; ^2^Older Adult Program, Fraser Health Authority, Surrey, BC, Canada V3R 7K1; ^3^Centre for Hip Health & Mobility, University of British Columbia, Vancouver, BC, Canada V5Z 1M9; ^4^School of Engineering Science, Simon Fraser University, Burnaby, BC, Canada V5A 1S6; ^5^Department of Family Practice, University of British Columbia, Vancouver, BC, Canada V5Z 1M9

## Abstract

**Objective:**

Identification of the factors that influence sedentary behaviour in older adults is important for the design of appropriate intervention strategies. In this study, we determined the prevalence of sedentary behaviour and its association with physical, cognitive, and psychosocial status among older adults residing in Assisted Living (AL).

**Methods:**

Participants (*n* = 114, mean age = 86.7) from AL sites in British Columbia wore waist-mounted activity monitors for 7 consecutive days, after being assessed with the Timed Up and Go (TUG), Montreal Cognitive Assessment (MoCA), Short Geriatric Depression Scale (GDS), and Modified Fall Efficacy Scale (MFES).

**Results:**

On average, participants spent 87% of their waking hours in sedentary behaviour, which accumulated in 52 bouts per day with each bout lasting an average of 13 minutes. Increased sedentary behaviour associated significantly with scores on the TUG (*r* = 0.373, *p* < 0.001) and MFES (*r* = −0.261, *p* = 0.005), but not with the MoCA or GDS. Sedentary behaviour also associated with male gender, use of mobility aid, and multiple regression with increased age.

**Conclusion:**

We found that sedentary behaviour among older adults in AL associated with TUG scores and falls-related self-efficacy, which are modifiable targets for interventions to decrease sedentary behaviour in this population.

## 1. Introduction

Physical activity (body movements that result in energy expenditure) and sedentary behaviour (periods of inactivity, typically while sitting or lying) are important markers of health and quality of life in older adults. An increase in physical activity has been shown to lessen functional decline [1, 2], decrease risk for falls and fall-related injuries [3, 4], and reduce the risk for obesity, hypertension and diabetes [5, 6] in older adults. Recent studies have shown that, independent of physical activity, decreases in the time spent in sedentary behaviour, and increases in the number of breaks in sedentary time, can have a positive effect on health outcomes [7, 8].

An accurate assessment of sedentary behaviour is important to inform the design of improved strategies to preserve and enhance the mobility of older adults. The design of improved strategies to reduce sedentary behaviour also requires an understanding of the modifiable factors that associate with sedentary behaviour. Physical, cognitive, and psychosocial status (depression and fear of falling) are important factors of successful aging and have been previously associated with sedentary behaviour [9]. Furthermore, previous research suggests that sedentary behaviour might be different between men and women [10] and that the level of physical activity is related to the use of mobility aids [11, 12].

Past research has relied on self-reported assessments of the time spent in sedentary activities during daily life, which suffer from limited accuracy (e.g., recall bias). For example, Harvey et al. [13] found that self-reported sedentary behaviour of older adults averaged 5.3 hours per day, well below values acquired with accelerometry, which averaged 9.4 hours per day. To address the inaccuracy of self-reports, wearable sensing technology (e.g., accelerometers) has emerged as a standard approach to objectively assess sedentary behaviour in older adults. Most studies have been conducted with community-dwelling older adults [14, 15], who wore an accelerometer for consecutive days, that classified their behaviour based on activity counts per minute. Using this method, Evenson et al. [15] found that community-dwelling older adults spend on average 8.5 hours (510 minutes) per day in sedentary behaviour.

Few studies have objectively investigated sedentary behaviour in older adults residing in Assisted Living (AL) sites (or retirement communities) [10, 16]. AL sites are increasingly popular, independent housing units that provide tenants with access to a level of care that is typically greater than that available in the community, but less than the skilled nursing care available in nursing home (or long-term care) settings. AL sites provide services (e.g., dining, laundry, housekeeping, and social and recreational programs) that allow older adults to remain independent as long as they are able to self-direct their own care. However, the level of physical and cognitive impairment in the AL population is typically greater than in older adults living in the community, which makes older adults living in AL more likely to be sedentary and less amenable to traditional tools (e.g., self-reports) [9].

An improved understanding of the prevalence and factors associated with sedentary behaviour in the growing AL population would facilitate the design of appropriate interventions to reduce sedentary behaviour in this setting. Accordingly, our goals in this study were to objectively determine (i) the prevalence of sedentary behaviour based on accelerometry and (ii) the associations between sedentary behaviour and independent measures of physical, cognitive, and psychosocial function in older adults residing in Assisted Living.

## 2. Methods

### 2.1. Participants

Older adults from 13 AL sites with publicly funded units located in the Fraser Health Authority (FHA) region of greater Vancouver were invited to enroll into this cross-sectional study [17]. Participants were eligible to participate if they were 65 years or older, could read and understand simple directions in English, and did not regularly use a wheelchair to move about. Sample size was selected to provide a statistical power of 80% to detect significant association between physical, cognitive, and psychosocial status and sedentary behaviour using multiple regression.

All participants provided written informed consent, and the study was approved by the research ethics boards of FHA and Simon Fraser University. A structured interview was used to determine participant age, length of stay in AL, highest level of education, habitual use of mobility aids, and number of health concerns (defined as physician-diagnosed medical conditions known to the participant, including arthritis, osteoporosis, hypertension, Parkinson's Disease, diabetes, stroke, or heart, kidney, lung, or liver disease).

### 2.2. Measure of Sedentary Behaviour

Participants were asked to wear an accelerometer-based activity monitor (GT1M, Actigraph) attached to a belt around their waist. The GT1M monitor has been commonly used and shown to be a valid tool for measuring physical activity and sedentary behaviour in young [18, 19] and older adults [14, 20]. The GT1M outputs a series of activity counts, which are quantitative measures of the intensity of the participant's physical activity over time. The activity counts depend only on the acceleration of the waist and do not rely on accurate step detection (e.g., pedometer), which can be challenging in frail older adults. The GT1M was initialized according to manufacturer specifications prior to being provided to participants. During this initialization, the sampling epoch (or independent sampling interval) was set to 10 seconds. Therefore, for each 10-second epoch, changes in acceleration were summed into a single activity count. For comparison to other studies, we summed each six consecutive (10 second epoch) activity counts to effectively yield activity counts for 1-minute epochs.

Each participant was instructed to wear the accelerometer during all waking hours for 7 consecutive days, put the sensor on immediately after they wake up, and remove it only for bathing or showering or prior to going to bed at night (to minimize the risk for disruptions in sleep due to discomfort from the sensor). Nonwear time was defined by an interval of at least 60 minutes of consecutive zero activity counts and removed from the analysis [21]. We only included in our analysis participants (*n* = 114) with three or more valid days of accelerometer data, where a valid day was defined by at least eight hours of wear.

The sensor data were analysed using MATLAB (The Mathworks) to classify the activity counts for each 1-minute epoch based on commonly used cut-points for older adults [14], where 0–99 counts represents sedentary behaviour, 100–1951 counts represents light physical activity, and more than 1952 counts represents moderate to vigorous physical activity. For each participant, we then calculated the percent of waking time spent in each of these three activity levels. Our primary outcomes were the percent of waking time spent in sedentary behaviour, the average number of sedentary bouts per day, and the average duration of each sedentary bout.

### 2.3. Ancillary Measures

We characterized physical function with the Timed Up and Go (TUG) and the Short Physical Performance Battery (SPPB) tests as described in [22, 23]. In the TUG, participants were instructed to rise from a chair, walk a distance of three meters, turn, walk back to the chair, and sit down. The test was scored as the time required to complete the task (recorded by a stop watch). In general, scores of 10 s or below indicate high functioning, while scores above 30 s indicate significant mobility impairment [24]. In the SPPB test, performance was scored between 0 and 4 in each of three timed components: (1) 4 meter timed walk; (2) static balance; and (3) sit-to-stand. A total score between 0 and 3 is typically regarded as severe limitation in physical function, 4–6 as moderate impairment, 7–9 as mild impairment, and 10–12 as minimal impairment.

We assessed cognitive function with the Montreal Cognitive Assessment (MoCA) [25]. Scoring on the MoCA ranges from 0 to 30, with a score ≤25 indicative of cognitive impairment [26]. We assessed psychosocial function with the 15-item Short Geriatric Depression Scale (GDS) [27], for which scores of 0–4 indicate no depression, 6–8 mild depression, 9–11 moderate depression, and 12–15 severe depression. We measured falls-related self-efficacy with the 14-item Modified Falls Efficacy Scale (MFES) [28, 29], which probes confidence in undertaking various activities without falling (e.g., getting dressed, taking a bath) on a scale of 0 (not confident at all) to 10 (completely confident). An overall MFES score (maximum score = 140) was calculated by averaging the scores for the 14 individual tasks.

### 2.4. Statistical Analysis

We used conservative nonparametric statistics to compensate for skewed distributions and reported values of measures of sedentary behaviour (and physical activity) with medians and interquartile ranges (IQR). We used Spearman's rank correlation to test whether measures of sedentary behaviour (percentage of time spent in sedentary behaviour, average number and duration of sedentary bouts) were associated with age, length of time in AL, number of reported health concerns, and scores on the TUG, SPPB, gait speed, MoCA, GDS, and MFES tests. We used the Mann–Whitney* U*-test to test for differences in sedentary behaviour between men and women, and between those who did and did not use a mobility aid. We used multiple linear regression models to examine how the variability in our primary outcomes was explained by the combination of physical function (TUG), cognitive function (MoCA), psychosocial function (GDS, MFES), age, sex, use of a mobility aid, and number of health concerns. All statistical analyses were conducted with a significance level of alpha = 0.05 using PASW Statistics (SPSS Inc.).

## 3. Results

### 3.1. Demographic Characteristics

**Figure 1:**
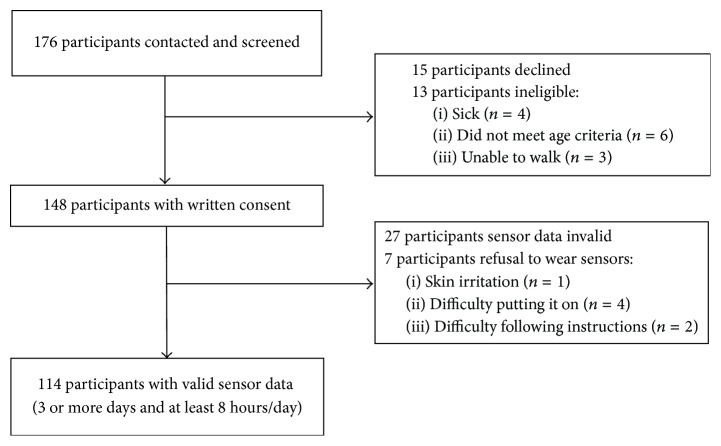
Participant recruitment and study design.

Of the 176 participants contacted to participate in this study, 13 were ineligible, 15 declined to participate, and 148 provided written informed consent (Figure 1). A total of 114 participants (85% women; mean age = 86.7 years, SD = 7.5) completed all tests and had valid accelerometer data (Table 1). On average, participants had lived in AL for 25.0 (SD = 17.6) months and reported 2.5 (1.4) health concerns. Close to one-quarter (23.7%) reported postsecondary education. In regard to the use of a mobility aid, 16% of participants reported not using a mobility aid, 72% reported habitual use of a walker, and 12% reported habitual use of a cane.

### 3.2. Test Scores from Physical, Cognitive, and Psychosocial Function Measures

The mean TUG score was 20.7 (SD = 10.0) seconds, with 6% of participants scoring below 10 seconds (Table 1). The mean SPPB score was 5 out of 12, with 98% of participants scoring below 10. On the MoCA, 85% of participants scored below 26, indicating some degree of cognitive impairment. Scores on the GDS ranged from 0 to 14 (out of 15), with 19% scoring above 5, indicating depressive symptoms. The mean MFES score was 8.0 (SD = 1.8) out of 10.

### 3.3. Sedentary Behaviour from Accelerometry

Participants wear the sensor on average for 6.2 days and 12.5 hours per day (nonwear time: 11.5 hours). The analysis revealed that participants put on the sensor at approximately 7:30 in the morning and took it off around 8:00 in the evening. Participants spent 86.9% of their waking time in sedentary behaviour, 12.9% in light physical activity, and 0.1% in moderate to vigorous physical activity (MVPA) (Table 2). In terms of absolute time, each day the average participant spent 10.9 hours (654 minutes) in sedentary behaviour, 1.6 hours (96 minutes) in light physical activity and 0.01 hours (1 minute) in MVPA. The average number of sedentary bouts per day was 51.5, and the average duration of a sedentary bout was 13.4 minutes.

### 3.4. Factors Associated with Sedentary Behaviour

Based on Spearman's rank correlation (Table 3), the percentage of waking time spent in sedentary behaviour significantly associated with scores on the TUG (*r* = 0.373, *p* < 0.001), SPPB (*r* = −0.282, *p* = 0.002), gait speed (*r* = −0.248, *p* = 0.008), and scores on the MFES (*r* = −0.261, *p* = 0.005), but not with age, length of stay in AL, number of reported health concerns, MoCA, or GDS. Similar associations were observed for the number and duration of sedentary bouts (besides scores on the GDS).

Based on the Mann–Whitney* U*-test (Table 4), the percent of waking time spent in sedentary behaviour was significantly greater in men than women (92.0% versus 86.4%, *p* = 0.008). Men had fewer bouts of sedentary behaviour, but the average duration of each bout was longer. Participants who used a mobility aid spent more waking time in sedentary behaviour than those who did not (87.7% versus 83.0%, *p* = 0.009) and exhibited fewer but longer sedentary bouts.

In our multiple linear regression models (Table 5), we included TUG (but not SPPB or gait speed) as a measure of physical function, since TUG associated most strongly with outcomes related to sedentary behaviour and correlated significantly with SPPB (*r* = −.659, *p* < 0.001) and with gait speed (*r* = −.763, *p* < 0.001). We found that a total of 23% of the variability in waking time spent in sedentary behaviour was explained by the combination of TUG score, age, and sex (*F*_8,105_ = 4.6, *p* < 0.001). An increase of 10 s in TUG score was associated with an increase of 2.3% in time spent in sedentary behaviour, while a 10-year increase in age was associated with a 1.9% increase in time spent in sedentary behaviour.

A total of 26% of the variability in number of sedentary bouts per day was explained by the combination of TUG score, sex, and MFES (*F*_8,105_ = 7.2, *p* < 0.001), and 34% of the variability in the average duration of sedentary bouts was explained by the combination of TUG score and sex (*F*_8,105_ = 5.6, *p* < 0.001).

## 4. Discussion

In this study, we objectively determined the prevalence of sedentary behaviour and its association with physical, cognitive, and psychosocial function among 114 tenants of AL, of mean age 87 years. We found that, on average, participants spent 87% of their waking hours in sedentary behaviour. Participants displayed a daily average of 52 bouts of sedentary behaviour, and the average duration of each sedentary bout was 13 minutes. On average, our participants spent 96 minutes of each day in light to moderate physical activity, and only one minute per day in moderate to vigorous physical activity (MVPA).

Our results support the growing evidence of the need for interventions to reduce sedentary behaviour (and increase physical activity) among older adults. In comparison to previous studies, we found that older adults in AL spend more time of their waking time in sedentary behaviour (87%) than it has been reported for community-dwelling older women (65%) [30] and men (72%) [31]. 87% was also higher than previously reported values of 71% for sedentary behaviour in older adults living in retirement communities [10, 16]. In terms of the time spent in MVPA, our participants were far below the physical activity guidelines for older adults of 150 minutes per week (21 minutes per day) of MVPA [32]. Official guidelines for sedentary behaviour are warranted, but do not exist for older adults yet [32, 33].

We also examined how patterns of sedentary behaviour associated with physical, cognitive, and psychosocial status. We found that participants who spent more time in sedentary behaviour had lower performances on tests of physical function (especially TUG, but also SPPB and gait speed) and had lower fall-related self-efficacy (MFES). Moreover, we found that participants with lower performances on tests of physical function, MFES, and GDS accumulated the sedentary time in fewer but longer sedentary bouts. Both physical capacity and falls-related self-efficacy are modifiable, and our findings are in agreement with Rosenberg et al. [9] and Van Lummel et al. [34], who reported similar associations. When compared to women, men spent 6% more of their waking hours in sedentary behaviour, and the average duration of sedentary bouts was longer in men than in women. Higher sedentary time in men compared to women, and fewer but longer bouts, was also reported by Bellettiere et al. [10]. We did not find that sedentary behaviour associated with cognitive status (MoCA). This finding is agreement with a previous study [9] where the authors used the Trail Making Test to assess cognitive status and also did not find an association with objectively measured sedentary time.

Furthermore, sedentary behaviour did not associate with demographic status, or number of health-related concerns. It is important to note that the variables we measured only explained 23% in the variability of time spent sedentary. This indicates that sedentary behaviours among older adults in AL can only be limitedly inferred from routine clinical measures, highlighting the need to measure sedentary behaviour through techniques such as accelerometry during daily life.

Our results show that sedentary behaviour in AL is significantly associated with physical function and falls-related self-efficacy. Future interventions may decrease sedentary behaviour in this population by specifically targeting functional movements and concern of falling. When combined with growing evidence of the negative health consequences of prolonged sedentary behaviour [7, 13], our results also highlight the need to develop strategies (e.g., brief exercise bouts) to break up sedentary bouts (especially among older men).

Our study had several limitations. First, despite our broad inclusion criteria, it is possible that healthier or more active older adults from AL sites were more likely to volunteer. However, there was considerable variability within our study sample in test scores on physical, cognitive, and psychosocial function measures, which argues against a possible selection bias. Furthermore, in our analysis we did not control for or excluded volunteers based on any specific medical conditions (e.g., Parkinson's disease, hypertension, and frailty), which might be factors that can be independently associated with sedentary behaviour in the AL population. Second, the activity counts measured by the accelerometer reflected body movements and did not capture static muscular exertions (such as resistance exercises, carrying a load, or bending over). Third, the Actigraph GT1M uses only a uniaxial accelerometer to sense vertical acceleration in contrast to the latest devices which use a triaxial accelerometer. Finally, we recruited participants from 13 AL sites, and while all were within the same health authority, physical activity patterns may have been influenced by environmental factors specific to each AL site. Future larger studies are warranted to examine the associations between sedentary behaviour and environmental factors such as the availability of exercise and recreational programs, physical layout, distance to amenities, and staff attitudes and motivation towards encouraging older adults to engage in daily activities (e.g., walking to the grocery store or hairdresser).

## 5. Conclusions

We examined the prevalence and factors associated with sedentary behaviour, as determined by accelerometry, among older adults residing in AL. On average, our participants spent 87% of their waking hours in sedentary behaviour. Sedentary behaviour associated significantly with modifiable measures of physical function and falls-related self-efficacy. This study adds important knowledge to inform future studies that employ targeted interventions to reduce sedentary behaviour in AL tenants.

## Figures and Tables

**Table 1:** Participant characteristics and test scores on physical, cognitive, and psychosocial function measures.

	Men (*n* = 17)	Women (*n* = 97)	Total (*n* = 114)
*Demographic characteristic*			
Age (mean (SD) in years)	85.7 (7.4)	86.8 (7.5)	86.7 (7.5)
Length of stay in AL (mean (SD) in months)	22.9 (15.4)	25.4 (18.0)	25.0 (17.6)
Postsecondary education (%)	31.6	21.7	23.7
Regular use of mobility aid (%)	88.2	83.5	84.2
Reported health concerns (mean (SD) number)	2.71 (1.36)	2.46 (1.39)	2.50 (1.38)
Hypertension (%)	52.9	55.2	54.9
Parkinson's Disease (%)	11.8	5.2	6.2
*Test score*			
Timed Up and Go (mean (SD) in seconds)	20.1 (9.5)	20.8 (10.1)	20.7 (10.0)
Short Physical Performance Battery (mean (SD) score out of 12)	4.94 (1.64)	5.28 (2.34)	5.23 (2.25)
Gait speed (mean (SD) in m/s)	0.71 (0.24)	0.75 (0.24)	0.75 (0.29)
Montreal Cognitive Assessment (mean (SD) score out of 30)	18.88 (6.44)	20.02 (5.14)	19.85 (5.33)
Short Geriatric Depression Scale (mean (SD) score out of 15)	3.8 (3.7)	3.1 (3.1)	3.2 (3.2)
Modified Falls Efficacy Scale (mean (SD) score out of 10)	7.8 (1.9)	8.1 (1.8)	8.0 (1.8)

**Table 2:** Percentage of time spent in sedentary behaviour, light physical activity and MVPA, and characteristics of sedentary bouts.

Accelerometer-derived variable	Median (IQR)
Percent of waking time spent in sedentary behaviour^a^	86.9 (8.6)
Percent of waking time spent in light physical activity^b^	12.9 (8.5)
Percent of waking time spent in MVPA^c^	0.1 (0.1)
Average number of sedentary bouts per day^d^	51.5 (22.4)
Average duration in minutes of sedentary bouts	13.4 (7.4)

*Notes*. ^a^Sedentary behaviour corresponds to <100 accelerometer counts/minute. ^b^Light physical activity corresponds to 100–1951 accelerometer counts/minute. ^c^Moderate to vigorous physical activity (MVPA) corresponds to >1952 accelerometer counts/minute. ^d^Sedentary bouts (1+) correspond to consecutive minutes involving <100 accelerometer counts/minute.

**Table 3:** Spearman's rank correlations between measures of physical, cognitive, and psychosocial function and accelerometry-derived measures of sedentary behaviour.

Independent variable	Dependent variable					
	Percentage of time sedentary		Number of sedentary bouts per day		Average duration of sedentary bouts	
	*r*	*p*	*r*	*p*	*r*	*p*
TUG	.373	<0.001^*∗∗*^	−.425	<0.001^*∗∗*^	.309	<0.001^*∗∗*^
SPPB	−.282	0.002^*∗∗*^	.382	<0.001^*∗∗*^	−.266	0.004^*∗∗*^
Gait speed	−.248	0.008^*∗∗*^	.244	0.009^*∗∗*^	−.206	0.028^*∗*^
GDS	.161	0.086	−.231	0.013^*∗*^	.184	0.049^*∗*^
MFES	−.261	0.005^*∗∗*^	.394	<0.001^*∗∗*^	−.277	0.003^*∗∗*^

*Notes*. (1) ^*∗*^*p* ≤ 0.05 level, ^*∗∗*^*p* ≤ 0.01 level; (2) no significant associations were observed for age, length of stay in AL, number of reported health concerns, and MoCA; (3) TUG: Timed Up and Go; SPPB: Short Physical Performance Battery; MoCA: Montreal Cognitive Assessment; GDS: Geriatric Depression Scale; MFES: Modified Fall Efficacy Scale.

**Table 4:** Associations between categorical variables of sex and use of mobility aid with accelerometry-derived measures of sedentary behaviour.

Accelerometer-derived variable	Sex			Use of mobility aid		
	Men (*n* = 17)	Women (*n* = 97)	*p*	No (*n* = 18)	Yes (*n* = 96)	*p*
Percent of waking time spent in sedentary behaviour	92.0 (8.0)	86.4 (7.8)	0.008^*∗∗*^	83.0 (11.2)	87.7 (8.3)	0.009^*∗∗*^
Average number of sedentary bouts per day	41.2 (35.3)	52.7 (22.1)	0.008^*∗∗*^	60.9 (25.0)	47.4 (22.3)	0.003^*∗∗*^
Average duration in minutes of sedentary bouts	17.1 (38.4)	12.3 (7.8)	0.002^*∗∗*^	10.9 (6.3)	13.8 (8.2)	0.012^*∗*^

*Notes*. (1) ^*∗*^*p* ≤ 0.05 level, ^*∗∗*^*p* ≤ 0.01 level.

**Table 5:** Results from multiple linear regression to predict accelerometry-derived measures of sedentary behaviour.

Dependent variable	Predictor variable	Unstandardized Beta	Standardized Beta	*p*
Percentage of time spent in sedentary behaviour	Sex	5.129	.233	0.007^*∗∗*^
	Age	0.187	.178	0.048^*∗*^
	TUG	0.226	.286	0.004^*∗∗*^

Number of sedentary bouts per day	Sex	−14.681	−.256	0.002^*∗∗*^
	TUG	−0.742	−.360	<0.001^*∗∗*^
	MFES	2.705	.239	0.011^*∗*^

Average duration of sedentary bouts	Sex	16.129	.342	<0.001^*∗∗*^
	TUG	0.645	.381	<0.001^*∗∗*^

*Notes*. (1) ^*∗*^*p* ≤ 0.05 level, ^*∗∗*^*p* ≤ 0.01 level; (2) the following variables were not significantly associated with the dependent variable in each of the 3 models: use of mobility aid, number of health concerns, MoCA, and GDS; (3) sex was coded as 0 = women and 1 = men.
